# ${\dot{V}O}_{2}$ kinetics and tethered strength influence the 200-m front crawl stroke kinematics and speed in young male swimmers

**DOI:** 10.3389/fphys.2022.1045178

**Published:** 2022-11-24

**Authors:** Kamil Sokołowski, Raul Filipe Bartolomeu, Tiago Manuel Barbosa, Marek Strzała

**Affiliations:** ^1^ Department of Water Sports, Faculty of Physical Education and Sport, University of Physical Education, Kraków, Poland; ^2^ Department of Sport Sciences and Physical Education, Instituto Politécnico de Bragança, Bragança, Portugal; ^3^ Department of Sports Sciences, Polytechnic of Guarda, Guarda, Portugal; ^4^ Research Center in Sports Sciences, Health and Human Development (CIDESD), Vila Real, Portugal

**Keywords:** adolescent swimming, oxygen uptake, tethered swimming, front crawl, biological age, kinematic indices

## Abstract

**Background:** The aim of this research was to examine the relationship between the fast component of oxygen consumption developed in 1-min ${\dot{V}O}_{2}$ and force indices both measured in tethered swimming test and to assess the influence of the gathered indices on speed and swimming kinematics in 200-m front crawl race.

**Methods:** Forty-eight male swimmers (aged 13.5 ± 0.9 years old) participated in this study. Testing included 1) 1-min all-out front crawl tethered swimming while oxygen consumption (breath by breath) and tethered forces were measured, 2) 200-m front crawl race-like swimming featuring kinematic analysis, and 3) biological age (*BA*) examination.

**Results:** During the 1-min all-out tethered swimming test, a linear increase in oxygen consumption was observed. There were moderate to high partial correlations between particular periods of seconds in the 1-min ${\dot{V}O}_{2}$ : 31–60, 41–60, and 51–60 and *F*_max_, *F*_*ave*_, and *I*_*ave*_ of tethered swimming, while 41–60 and 51–60 ${\dot{V}O}_{2}$ were moderately to highly interrelated with all the swimming speed indices and *SI*. The swimming speed indices significantly interplayed with *SL*, *SI*, *F*_max_, *F*_*ave*_, and *I*_*ave*_. Partial correlations were computed with *BA* control.

**Conclusion:** The ability of reaching a high level of ${\dot{V}O}_{2}$ fast is essential for a swimmer’s energy production at short- and middle-distance events. Reaching a high level of ${\dot{V}O}_{2}$ significantly determines tethered strength and swimming kinematics. The level of ${\dot{V}O}_{2}$ influences the maintenance of a proper pulling force and the stroke technique of front crawl swimming in young male swimmers.

## Introduction

The ability to increase energy production is considered crucial in various sports, even in swimming where high velocities cause relatively high energy cost of movement. Thus, it is necessary among athletes of different age groups to develop either aerobic or anaerobic metabolic pathways of energy production. This begins with proper and adequate training from early prepubertal age and continues further with aging, while controlling the maturation level of the swimmer (Balyi and Way, 2009; Lätt et al., 2009). The contribution of energy pathways in swimming events is varied and depends on the duration of the race (Olbrecht, 2000). The 200-m front crawl, for example, is a race which requires a high involvement of aerobic and anaerobic pathways of energy production (Gastin, 2001).

The aerobic energy system participates in the overall energy production right from the beginning of the all-out effort, and the oxygen uptake almost reaches its maximum level within 60 s of exercising (Gastin and Lawson, 1994; Serresse et al., 1988; Strzała and Tyka 2009). It has been stated that the maximal oxygen uptake $\left({\dot{V}O}_{2}\max\right)$ assesses the ability in developing and maintaining high speed of sprint swimmers in efforts lasting about 60 s (Ribeiro et al., 2015; Hellard et al., 2018). According to the data presented by Figueiredo et al. (2011), even in 200-m front crawl race, the aerobic pathway engages fast in providing energy for muscle work within half of the race, while at the third (long course) lap, aerobic metabolism provides for around 80% of all energy production. Among swimmers of different age groups, in the 200-m event, the aerobic contribution has been estimated to be 72% (Zamparo et al., 2000) or even 78.6% (Sousa et al., 2011). However, the contribution of the aerobic pathway of energy production in swimming at short and middle distances seems to have been underestimated over the past years (Peyrebrune et al., 2014). Rodriguez et al. (2003) have reported that swimmers not only reached 92.3% of their ${\dot{V}O}_{2}\max$ in the 100-m events but also exhibited ${\dot{V}O}_{2}$ kinetics that was significantly faster in the 100-m race than in the 400-m one. Their results highlight the significance of fast oxygen kinetics especially while competing in short races, such as the 100-m ones. Despite the existence of research on the relationship between oxygen consumption and swimming performance, there is a need to refresh (Costill et al., 1985) and further investigate the fast component of ${\dot{V}O}_{2}$ kinetics, i.e., the abrupt oxygen delivery to the body in short- to medium-term exercising periods. Moreover, there is a knowledge gap on the influence and dependence of this type of cardiorespiratory efficacy, present in most swimming races, on the ability to generate propulsion force and stroke kinematics.

In swimming, the examination of specific strength abilities is deemed as a key factor when performing an evaluation. For this purpose, swimming tethered tests are often conducted in adults (Kjendlie and Thorsvald, 2006) and swimmers of other age groups (Amaro et al., 2014). Several studies have confirmed a strong relationship between tethered swimming tests (30–120 s) and short-to-middle distance swimming performances (Morouço et al., 2012; Santos et al., 2016).

Biomechanical indices such as stroke length (*SL*), stroke rate (*SR*), and stroke index (*SI*) are significant predictors of young swimmers’ performance (Lätt et al., 2009) and are directly related to swimming efficiency (Geladas et al., 2005). The literature reports that strength preparation and a well-developed oxygen system should cause better stroke kinematics in terms of the ability to maintain proper SR and SL along the race (Costill et al., 1985; Sokołowski et al., 2021). Given these premises, the aim of this research was threefold: 1) to examine the relationship between the fast component of oxygen consumption and tethered swimming force production, 2) to examine the relationship between the fast component of oxygen consumption and 200-m front crawl race kinematics, and 3) to assess the relationship between 200-m front crawl race swimming kinematics and performance. It is hypothesized that there would be a significant relationship between oxygen uptake, tethered swimming force, stroke kinematics, and the performance indices.

## Materials and methods

### Participants

Forty-eight young male swimmers [13.5 ± 0.9 years old; 14.55 ± 1.66 years of biological age (*BA*)] participated in this study. They were recruited as swimmers with the highest performance level in their age category from the Polish region of Krakow and were at the fifth threshold in the Ruiz-Navarro et al. (2022) classification of competitive level. Participants presented swimming levels which resulted in a mean value of 350.32 ± 60.22 FINA points for the 200-m front crawl race. All participants were clinically healthy and held a license from the Polish Swimming Federation. All swimmers had been through 4–5 years of systematic swimming at the time of conducting this research, encompassing at least 10 sessions per week and had taken part in national-level competitions and national swimming championships for their age group.

### 1-min Tethered swimming test

**FIGURE 1:**
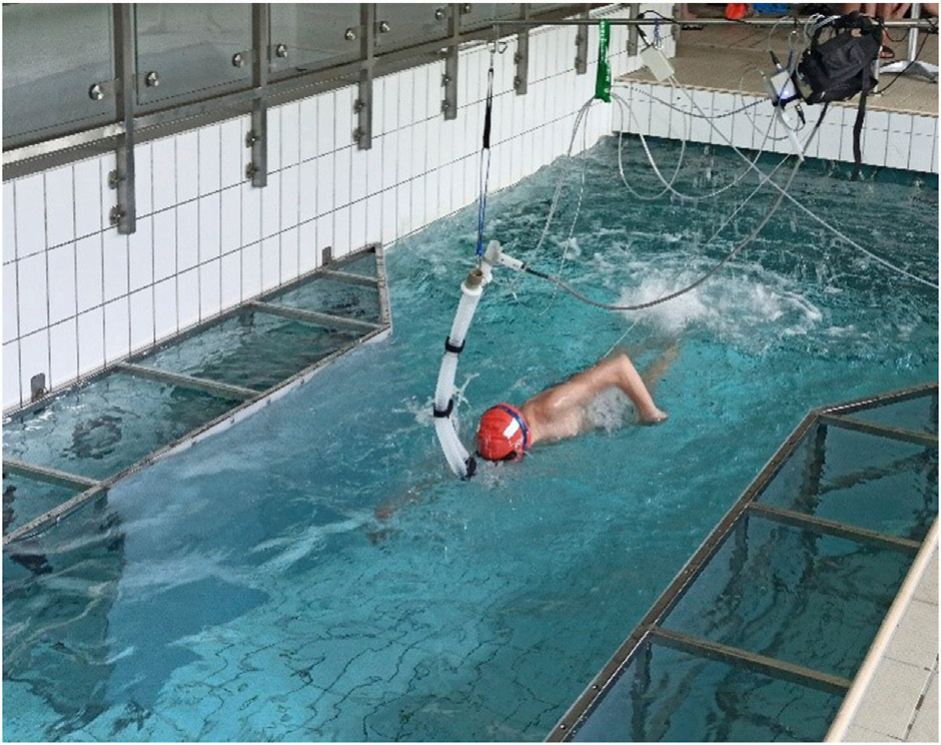
1-min tethered swimming test.

A tethered swimming test (Figure 1) in a laboratory-controlled environment (temperature and humidity) was conducted. The test consisted of a single bout of 1-min duration of all-out freestyle tethered swimming and was performed in a flume in still water. With due advance notice, the swimmers were asked to rest the day before the test and maintain their daily diet. Before entering the pool, they were informed about the testing procedure and then underwent a 1000-m in-water warm-up, as before any competition. After the warm-up and before the test, they swam for 1 min in the flume at a slow pace, fully equipped with the testing apparatus for adjusting to the testing conditions. At this time, they got the possibility to familiarize with the specific environment of the flume and potential inconveniences of using the breathing apparatus and tethered swimming. After the initial 1 min of familiarization, the scientist conducting the test received feedback from the participant. To signal the beginning and ending of the test, a whistle was used. For the last minutes of warm-up and the test itself, the swimmers were asked to breathe only through the mouthpiece and avoid losing their nose clip. This procedure is similar to their training sessions done using a snorkel. The swimmers were equipped with a respiratory valve system that featured an ergospirometer (Start 2000 MES, Poland). The valve system was attached to a rod-like construction just above the swimmer’s head. During the duration of the test, the expired air was analyzed continuously (breath by breath) (Ergo 2000M software MES, Poland) and data were saved for further analysis. This has been proved to be a reliable method of calculating oxygen uptake in swimming (Neiva et al., 2017; Ribeiro et al., 2015; Sousa et al., 2011).

From the collected data, the following indices were computed: 1) average oxygen consumption from the first 30 s of the test (1–30 ${\dot{V}O}_{2}$ , l∙min^−1^), 2) average oxygen consumption from the last 30 s of the test (31–60 ${\dot{V}O}_{2}$ , l∙min^−1^), 3) average oxygen consumption from the last 20 s of the test (41–60 ${\dot{V}O}_{2}$ , l∙min^−1^), 4) average oxygen consumption from the last 10 s of the test (51–60 ${\dot{V}O}_{2}$ , l∙min^−1^), and 5) oxygen consumption from the total test duration (1–60 ${\dot{V}O}_{2}$ , l∙min^−1^).

Additionally, the participants wore a nylon waist belt, connected by a 3.7 m steel cable to a load cell (ZPS5-BTU-1kN, Poland) which was fixed on a steel pole (the fixing point is 0.49 m above the water surface). Data were recorded by the load cell at 100 Hz and transferred to a computer software program for further analysis (MAX6v0M software, Poland). Three parameters were calculated over a 60-s recording time: 1) maximum value of force (*F*_max_, N); 2) average value of force in the entire test (*F*_*ave*_, N) and in the first and second 30-s parts: *F*_*ave 0-30*_, *F*_*ave 30-60*_, N; and 3) average impulse per single cycle (*I*_*ave*_, N·s^−1^) which is defined as the integral of force over a period of time containing all full cycles divided by the number of completed cycles:

$$I_{ave}=\frac{\int_{t_{0}}^{t_{1}}Fdt}{n}$$ (1)

where *t*_*0*_ is the beginning of the first full cycle and *t*_*1*_ is the ending of the last full cycle in the 60-s period. Tethered swimming has been described as a reliable method to assess swimming force production (Kjendlie and Thorsvald, 2006; Psycharakis et al., 2011; Amaro et al., 2014).

### 200-m Front crawl race

The 200-m all-out test was carried out in a 25-m swimming pool that meets the International Swimming Federation (FINA) requirements. Before the race, the swimmers completed a 1000-m warm-up just like in competitions. Each trial was performed by three to four swimmers in order to mimic competition conditions. The final and split times of each trial were measured with an automatic timing device (Omega, Switzerland; OCP5, StartTime V). All trials were recorded with a camera at 50 Hz framing (GC-PX100BE, JVC, Japan).

The velocity of the part of the race containing the first 10-m start zone as well as start, turn, and finish (which resulted in 115 m) was calculated as *V*_*STF*_ (m·s^−1^). The surface swimming velocity, i.e., the velocity over the effective clean swimming distance (85 m) was deemed *V*_*surf*_ (m·s^−1^). The times for separate sectors were measured when the swimmer's head crosses the imaginary line linking the markers at both sides of the pool. The 200-m front crawl velocity (*V*_*total200*_, m·s^−1^) was defined as 200 divided by the final time of the race. The video footage, placement of the cameras and markers, video analysis, and computation of the basic kinematic parameters were performed analogically to the ones described in the literature (Sokołowski et al., 2021), but in this study, a swimming distance twice as long was considered.

### Kinematic parameters

For the kinematic analysis, the stroke rate (*SR*), stroke length (*SL*), and stroke index were calculated. The *SR* was defined as the number of full stroke cycles performed within a unit of time (in cycles per minute) and was calculated by video analysis of three consecutive stroke cycles (intraclass correlation of 0.99, 95% CI = 0.960–0.997). The *SL* was defined as the horizontal distance that the body travels during a full stroke cycle and was calculated as

$$SL=\frac{v}{SR}$$ (2)

where *SL* (in m) is the stroke length, *v* is the swimming velocity, and *SR* is the stroke rate. Finally, the SI was deemed as an overall swimming efficiency estimator and computed as

SI=SL∙v (3)

where *SI* (in m^2^∙s^−1^) is the stroke index, *SL* is the stroke length, and *v* is the swimming velocity.

### Biological age

**FIGURE 2:**
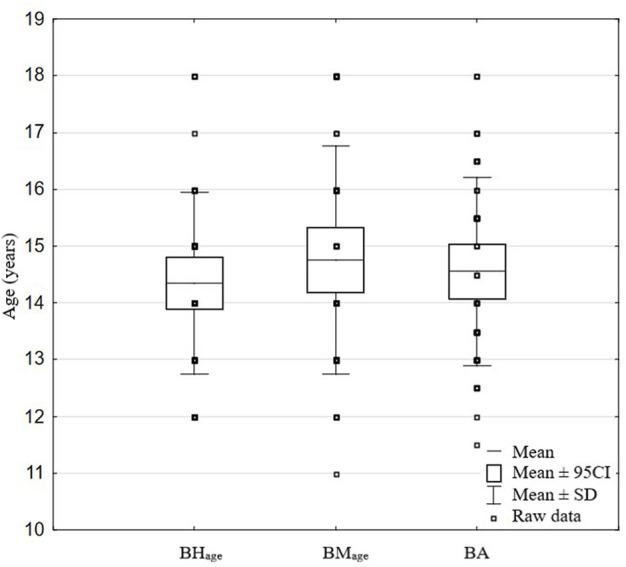
Average data of *BH*_*age*_, *BM*_*age*_, and *BA*.

Examination of the participants in terms of *BA* was conducted by an experienced anthropologist and calculated as

$$BA=\frac{\left(BHage+BMage\right)}{2}$$ (4)

where *BHage* is the age obtained from the percentile charts based on the participant’s body height and *BMage* is the age obtained from the percentile charts based on the participant’s body mass. The growth charts by the Children’s Memorial Health Institute, which are standardized and validated for the Polish population, were used (the 50th percentile was used to align the height and mass with age). Additionally, pubertal development was assessed. The Tanner stages based on pubic hair scale were estimated (Bornstein, 2018). The great variety of biological maturation levels in the adolescent groups at the same calendar age causes great differences in muscle mass and aerobic and anaerobic capacities of swimmers. Because of differences in maturation specific water abilities of swimmers and specific testing could be less correlated with swimming performance than simple general tests as isometric force or counter movement jump (Garrido et al., 2012; Strzała et al., 2019). *BA* may cause bias in the statistical analysis and conclusions. The use of partial correlation statistics with age control helps limit the strong influence of *BA* in the effects of statistical calculations. The data used in biological age calculation are presented in Figure 2.

### Statistical analysis

**FIGURE 3:**
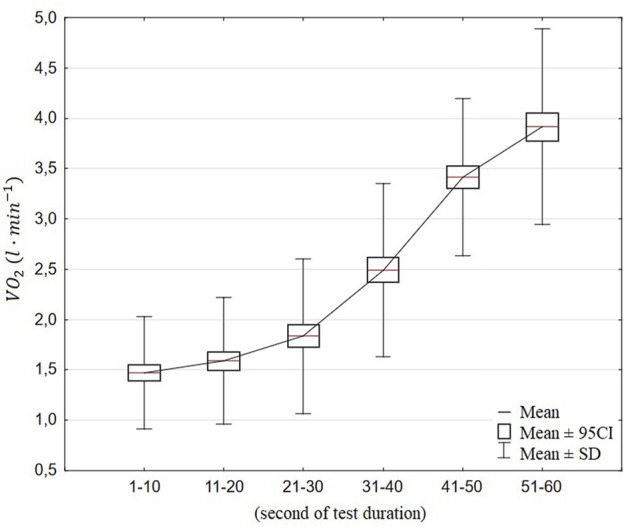
Average oxygen consumption of all participants, in 10-s periods, during the 1-min tethered swimming test.

The values are presented as mean ± standard deviation. The normality of the data was checked with the Kolmogorov–Smirnov test. In oxygen consumption averaged per 10-s periods, the trend that was most suitable for the gathered data (Figure 3) was identified. The paired-sample t-test was used to compare the values of the average tethered swimming force of the first and second parts of the 1-min tethered swimming test. To identify the relationship between all the variables and swimming velocities in the 200-m front crawl, partial correlations controlled for *BA* were computed for

1) oxygen consumption and force indices;
2) oxygen consumption, swimming speed variables, and kinematic indices; and
3) swimming speed variables and kinematic and force indices.

The magnitude of the correlations was determined using the modified scale by Hopkins (2000)—trivial: r ≤ 0.1; low: 0.1 < r ≤ 0.3; moderate: 0.3 < r ≤ 0.5; high: 0.5 < r ≤ 0.7; very high: 0.7 < r ≤ 0.9; nearly perfect: r > 0.9; and perfect: r = 1.

## Results

The data shown in Figure 3 represent the increase in oxygen consumption in the 1-min all-out tethered swimming test, in 10-s periods. The analysis of variance revealed significant differences between values measured every 10 s (F = 164,9, *p* < 0.01). Further trend analysis indicates the linear trend as the best adjusted to the collected data (F = 289,44, *p* < 0.01).

There were moderate to high correlations between 31–60 ${\dot{V}O}_{2}$ , 41–60 ${\dot{V}O}_{2}$ , and 51–60 ${\dot{V}O}_{2}$ and all the swimming force indices (*F*_max_, *F*_*ave*_, *I*_*ave*_). Low correlations were observed between *F*_max_, *I*_*ave*_, and 1–60 ${\dot{V}O}_{2}$ (Table 1). A significantly higher average of tethered force was noted in the first 30-s duration of the test: *F*_*ave 0-30*_ 85.41 ± 21.41 N *vs F*_*ave 30-60*_ 67.12 ± 15.22 (t = 14.77; df = 47; *p* ≤ 0.0000).

**TABLE 1:** Partial correlations controlled for *BA* between oxygen consumption and force indices from the tethered swimming test.

	*F*_max_ (N)	*F*_*ave*_ (N)	*I*_*ave*_ (N·s^−1^)
	250.24 ± 58.39	74.90 ± 20.63	101.93 ± 23.48
1–30 ${\dot{V}O}_{2}$ (l∙min^−1^)	0.167	0.053	0.134
1.68 ± 0.59			
31–60 ${\dot{V}O}_{2}$ (l∙min^−1^)	0.296*	0.363**	0.372**
3.30 ± 0.76			
41–60 ${\dot{V}O}_{2}$ (l min^−1^)	0.395**	0.494**	0.502**
3.65 ± 0.81			
51–60 ${\dot{V}O}_{2}$ (l min^−1^)	0.482**	0.516**	0.559**
3.92 ± 0.97			
1–60 ${\dot{V}O}_{2}$ (l min^−1^)	0.285*	0.245 *p = 0.054*	0.290*
2.55 ± 0.59			

*p ≤ 0.05; **p ≤ 0.01.

The 41–60 ${\dot{V}O}_{2}$ and 51–60 ${\dot{V}O}_{2}$ were moderately to highly correlated with all the swimming speed indices and *SI. V*_*surf*_ was also significantly correlated with 1–30 ${\dot{V}O}_{2}$ (Table 2). There was a positive correlation between *SL* and 51–60 ${\dot{V}O}_{2}$ *.*

**TABLE 2:** Partial correlations controlled for *BA* between oxygen consumption indices from the tethered swimming test, and swimming speed variables and kinematic indices from the 200-m front crawl race.

	*V*_*total200*_	*V*_*surf*_	*V*_*STF*_	*S*	*SL*	*SI*
	(m·s^−1^)	(m·s^−1^)	(m·s^−1^)	(cycles∙min^−1^)	(m)	(m^2^∙min^−1^)
	1.40 ± 0.09	1.34 ± 0.09	1.46 ± 0.10	41.68 ± 4.52	1.93 ± 0.24	2.53 ± 0.42
1-30 ${\dot{V}O}_{2}$ (l∙min^−1^)	0.187	0.299*	0.106	0.080	0.076	0.206
1.68 ± 0.59						
31-60 ${\dot{V}O}_{2}$ (l∙min^−1^)	0.294	0.311	0.288	-0.083	0.206	0.283
3.30 ± 0.76						
41-60 ${\dot{V}O}_{2}$ (l∙min^−1^)	0.463*	0.428*	0.487*	-0.136	0.310	0.412*
3.65 ± 0.81						
51-60 ${\dot{V}O}_{2}$ (l∙min^−1^)	0.640**	0.584**	0.666**	-0.119	0.393*	0.539**
3.92 ± 0.97						
1-60 ${\dot{V}O}_{2}$ (l∙min^−1^)	0.242	0.311	0.201	-0.007	0.155	0.255
2.55 ± 0.59		*p = 0.075*				

*p ≤ 0.05; **p ≤ 0.01.

Regarding the swimming speed and kinematic variables, the strongest relationships were observed between *SI* and *V*_*total200*_ and *V*_*surf*_ and *V*_*STF*_. The swimming speed was also moderately correlated with *SL*, *F*_max_, *F*_*ave*_, and *I*_*ave*_ (Table 3).

**TABLE 3:** Partial correlations controlled for *BA* between swimming speed variables and kinematic indices from the 200-m front crawl race and the force indices from the tethered swimming test.

	*SR*	*SL*	*SI*	*F*_max_	*F*_*ave*_	*I*_*ave*_
	(cycles∙min^−1^)	(m)	(m^2^∙s^−1^)	(cycles∙min^−1^)	(cycles∙min^−1^)	(cycles∙min^−1^)
*V*_*total200*_	0.168	0.325*	0.680**	0.341**	0.321**	0.406**
*V*_*surf*_	0.229	0.301*	0.692**	0.321**	0.408**	0.387**
*V*_*STF*_	0.103	0.337*	0.644**	0.355**	0.411**	0.407**

*p ≤ 0.05; **p ≤ 0.01.

As a supplement to the results, it was decided to present the level of selected oxygen uptake and strength indicators, measured in the 1-min test, followed by the kinematics of 200-m front crawl in relation to *BA* (Table 4). It could be observed that oxygen uptake and strength abilities continuously improve with higher *BA*. There was also a general increase in values of stroke kinematics through the years of *BA.*

**TABLE 4:** Average values of oxygen uptake, tethered swimming, and kinematic indices of 200-m front crawl calculated for biological age.

*BA* (years)/number of participants (n)	51–60	1–60	*F*_*ave*_	*I*_*ave*_	*SR*	*SL*	*SI*	*V*_*total200*_
	${\dot{V}O}_{2}$	${\dot{V}O}_{2}$	(N)	(N·s^−1^)	(c∙min^−1^)	(m)	(m^2^∙min^−1^)	(m·s^−1^)
	(l∙min^−1^)	(l∙min^−1^)						
11 (n = 1)	2.03	1.33	61.9	89.41	38.88	2.12	2.89	1.43
12 (n = 5)	3.10	1.95	47.31	74.05	45.81	1.72	2.21	1.36
13 (n = 15)	3.42	2.39	65.18	88.31	42.38	1.87	2.45	1.38
14 (n = 5)	3.47	2.38	70.82	98.14	38.62	1.96	2.34	1.31
15 (n = 9)	4.54	2.77	88.6	117.78	41.70	1.99	2.69	1.45
16 (n = 7)	4.41	3.00	82.73	112.44	40.96	1.93	2.49	1.40
17 (n = 4)	4.75	2.91	96.65	123.83	40.09	2.13	2.94	1.48
18 (n = 2)	5.56	3.13	100.95	137.69	40.82	2.08	2.90	1.51

Table 5 shows 200-m front crawl kinematics by each 50-m lap.

**TABLE 5:** Average values of kinematic indices for each 50-m lap of 200-m front crawl.

	I 50	II 50	III 50	IV 50
*SR* (cycles∙min^−1^)	42.91 ± 5.49	40.44 ± 4.63	39.99 ± 4.89	43.93 ± 4.87
*SL* (m)	1.97 ± 0.29	1.92 ± 0.24	1.90 ± 0.24	1.85 ± 0.23
*SI* (m^2^∙min^−1^)	2.75 ± 0.54	2.46 ± 0.41	2.40 ± 0.41	2.49 ± 0.42
*V*_*surf*_ (m·s^−1^)	1.45 ± 0.11	1.29 ± 0.09	1.26 ± 0.10	1.32 ± 0.09

## Discussion

Regarding the analysis of ${\dot{V}O}_{2}$ kinetics, an instantaneous and sudden increase was observed along the 1-min all-out tethered swimming. Despite the increase in ${\dot{V}O}_{2}$ which could be characterized as a linear increase, the slopes in both initial and final segments of the 1-min consumption were noticeably lower than the one observed at the middle (Figure 3). Slower oxygen uptake at the beginning of the test may be associated with the use of high-energy phosphocreatine resources and yet low ventilation ( $\dot{V}$ *E*); the final slowdown in ${\dot{V}O}_{2}$ growth is from reaching a peak and increasing fatigue. This study revealed a significant influence of ${\dot{V}O}_{2}$ (mainly 41–60 ${\dot{V}O}_{2}$ and 51–60 ${\dot{V}O}_{2}$ ) on 200-m front crawl race swimming speed, swimming kinematic indices, and tethered force indices. A highly developed fast ${\dot{O}}_{2}$ supply to working muscles (represented by 51–60 ${\dot{V}O}_{2}$ ) is significantly related to strength (0.482 ≤ *r* ≤ 0.559, *p* ≤ 0.01). This strength in swimming is expressed as the ability to produce propulsive force, which is later translated into higher stroke efficiency and thus better swimming economy (51–60 ${\dot{V}O}_{2}$ *vs SI*, *r =* 0.539, *p* ≤ 0.01). Similarly, the higher energy demand connected with 51–60 ${\dot{V}O}_{2}$ translated into significantly higher *V*_*surf*_ (r = 0.584, *p* ≤ 0.05), which depended on proper swimming economy, due to the relationship between *V*_*surf*_ and *SI* (r = 0.692, *p* ≤ 0.01) and *I*_*ave*_ (0.387, *p* ≤ 0.01).

This study noted a relationship between 51–60 ${\dot{V}O}_{2}$ and the overall performance in 200-m front crawl (r = 0.640, *p* ≤ 0.01) which is in tandem with the results of Rodriguez et al. (2003), where a correlation between ${\dot{V}O}_{2}$ peak values and the performance at 100 m (r = 0.787, *p* ≤ 0.05) and 400 m (r = 0.752, *p* ≤ 0.05) was observed. The reason for a weaker correlation in our study could be the longer period considered for the mean ${\dot{V}O}_{2}$ calculation. We used 10-s periods, while Rodriguez et al. (2003) used 5-s periods. The breath-by-breath acquisition technique can induce a significant variability on acquired ${\dot{V}O}_{2}$ values, and different sampling periods might produce different outcomes. Moreover, our quite restrictive statistical calculations (including *BA* control) could also play a role in that difference. In comparison to the results of Sousa et al. (2011), which showed a positive correlation between 200-m front crawl swimming speed and ${\dot{V}O}_{2}$ peak (r = 0.69, *p* = 0.03), our partial correlation was somewhat slightly lower (r = 0.640, *p* ≤ 0.01). Nevertheless, these researchers found high ${\dot{V}O}_{2}$ values right after the first 50 m that swimmers could almost maintain for the 200-m effort. Researchers have put forward that the need for oxygen in the muscles triggers an instantaneous and sudden increase in $O_{2}$ uptake from the very beginning of the exercise (Ribeiro et al., 2015; Hellard et al., 2018). Maybe the highest peak of $O_{2}$ uptake could be reached even faster in our study and show faster kinetics in young athletes, but because it is in swimming, the aim of racing (also through the test) is to withstand the pace as much as possible until the end of the race. Nevertheless, in our research, we recorded a positive distribution of average tethered swimming force (*F*_*ave 0-30*_ 85.41 ± 21.41 N *vs F*_*ave 30-60*_ 67.12 ± 15.22 N). The question here is how speedily and individually for a competitor, should a race be open to young 13-year-old swimmers in order to allow for the proper engagement of the fast component of oxygen consumption. It is known that positive pacing, or rather starting a race too speedily, can cause excessive fatigue, low oxygen distribution, and lactic acidosis in the skeletal muscles, which slow down energy production in the aerobic pathway. It may also be due to fatigue of the chest breathing muscles during the second part of the 200-m distance (Gastin and Lawson, 1994).

It can be stated that for high aerobic capacity, the fast development of high level of $O_{2}$ supply is crucial while performing middle distance events such as the 200-m front crawl. For this purpose, the 1-min tethered swimming test seems to be appropriate in examining the ability to supply $O_{2}$ to the swimmer’s muscles to produce propulsion. Serresse et al. (1988) who examined the maximum 90-s ergocycle test observed that the highest ${\dot{V}O}_{2}$ values occurred at about 60 s into the test. Similar to our study, their results have shown a linear increase in oxygen uptake up to 60 s into the test. Gastin and Lawson (1994) stated that 30–60 s of maximum effort could be enough to reach up to 90% of athletes’ ${\dot{V}O}_{2}$ max. Ribeiro et al. (2015) claimed that if the majority of the swimming races are 50, 100, and 200 m, performed at high speeds, examining the ${\dot{V}O}_{2}$ max at low intensities has limited application in the evaluation of the swimmer's conditioning. Alves et al. (2011) suggested that faster kinetics during the initial phase of ${V\dot{O}}_{2}$ max testing is directly related to a better performance at middle-distance events in swimming. Based on this reasoning, one could suggest that middle-distance swimmers should undergo long, high-intensity aerobic repeated sprints in training sessions.

Regarding tethered force production, in the present study, a significant positive correlation was found between all indices and 200-m front crawl speed (0.321 ≤ *r* ≤ 0.411, *p* ≤ 0.01). Other authors have reported similar findings: Santos et al. (2016) have noted a positive correlation (0.61, *p* < 0.001) between the peak force of the 2-min tethered swimming test and clean velocity of 200-m front crawl race, while Morouço et al. (2012) showed a very strong relationship between average pulling force, peak force, and 200-m front crawl velocity (r = 0.94 and r = 0.93, respectively, *p* < 0.01). Again, controlling for *BA* and longer test duration could be the reasons for weaker correlations in our study.

Our study showed great diversity in *BA* (Figure 2; Table 4). It is therefore a practical example of emphasizing the need for each trainer to adapt their training in relation to the *BA* of their swimmers. If this is the case, even the most gifted swimmers with delays in relation to *BA* are often frustrated by worse athletic performance when compared to their calendar peers, and in consequence, they overtrain trying to catch up to the others, get disappointed, then quit their swimming training. On the other hand, swimmers more advanced in relation to *BA* have the potential to develop through more individualized, intense training.

Based on the high correlation between 51–60 ${\dot{V}O}_{2}$ and *SI* found in the present study (r = 0.539, *p* ≤ 0.01), we can state that peak oxygen consumption determined the rate of transfer from chemical energy to mechanical energy, thus leveling up the stroke kinematics of the swimmers. This finding backs up the results by Sánchez and Arellano (2002), where the *SI* was found to be higher in international-level swimmers than their national-level counterparts in all swim strokes. Barbosa et al. (2013) proposed a multidisciplinary model of swimming performance predictors where the *SI* plays a significant role. In a study by Costill et al. (1985), the predictability of ${\dot{V}O}_{2}\max$ at freestyle was reported to increase significantly when the *SI* was included in the multiple regression analysis of an approximate 400-m swim. The multiple regression models prepared by Mezzaroba and Machado (2013) revealed that in young male swimmers, the *SI* at the 200-m front crawl race explained 76% of the performance. In the study by Nasirzade et al. (2015), 200-m front crawl performance of young swimmers was strongly related to the *SL* and *SI* (r = −0.79 and r = −0.72, *p* < 0.01, respectively). The mentioned studies are in tandem with our results where *SI* presented the highest positive correlation with all 200-m front crawl variables (0.644 < *r* ≤ 0.692, *p* ≤ 0.01). This very high percentage of share of the *SI* in performance in the abovementioned studies is also because of its link to performance itself, because the stroke index contains the speed (according to the formula: SI=SL∙v ).

The present study, analyzing the relationship between the aerobic conditioning level, force production, and stroke kinematics is in accordance with the one study found in the literature on this matter, where Costill et al. (1985) identified interrelationships between oxygen uptake, energy cost of swimming, and stroking economy (*SI*). In our study, we found moderate to high correlations between 31–60 ${\dot{V}O}_{2}$ , 41–60 ${\dot{V}O}_{2}$ , and 51–60 ${\dot{V}O}_{2}$ and *F*_max_, *F*_*ave*_, and *I*_*ave*_. Low correlations were observed between *F*_max_, *I*_*ave*_, and 1–60 ${\dot{V}O}_{2}.$ It could be stated that the ability to generate the pulling force is directly and positively related to the fast $O_{2}$ supply which is linked with the endurance of the swimmer in terms of aerobic energy production and also lactate utilization or turnover to ATP (Greenwood et al., 2008).

## Conclusion

In the 1-min all-out effort, a sudden increase in oxygen uptake was observed, with swimmers reaching high levels of ${\dot{V}O}_{2}$ by the end of the tethered test. This fast ability of reaching high ${\dot{V}O}_{2}$ and trainability of this physiological variable is essential for fitting an appropriate pacing in middle-distance racing and must be an important aspect of 13-year-old swimmers’ conditioning and of the older age groups too, in relation to their *BA*. Furthermore, it is suitable for the physiological preparation for 200-m front crawl performance and can be useful as a predictor of the swimmer's endurance. The high intensity ${\dot{V}O}_{2}$ testing used in the present study is appropriate for predicting sprint (100-m) and middle-distance swimming events performed at high speeds. There is a relationship between the fast-developed 1-min high-level oxygen uptake and the tethered strength abilities and high-speed swimming. The fast $O_{2}$ supply is crucial for maintaining a proper pulling force and stroke technique.

## Data Availability

The raw data supporting the conclusions of this article will be made available by the authors, without undue reservation.
